# Knowledge, Attitude and Practice Study of Health Risks Among E-waste Recyclers in Delhi

**DOI:** 10.5696/2156-9614-11.29.210306

**Published:** 2021-03-02

**Authors:** Diksha Singhal, Tanica Lyngdoh, Poormima Prabhakaran

**Affiliations:** 1 Indian Institute of Public Health-Delhi, Public Health Foundation of India, Institutional Area Gurugram, Delhi, India; 2 Centre for Environmental Health, Public Health Foundation of India, Institutional Area Gurugram, Delhi, India

**Keywords:** e-waste, informal recycling, health risks, occupational hazards

## Abstract

**Background.:**

India is the fifth biggest producer of e-waste in the world, discarding 1.7 million tons in 2014. E-waste gets recycled mainly in the informal sector which means activities are largely unaccounted for. Hazardous chemicals and metals are released during recycling processes (lead (Pb) being the most common). Compared to other developing countries, there are few studies from India on the awareness of recyclers of health risks related to e-waste recycling.

**Objectives.:**

The aim of the present study was to assess the knowledge, attitudes, and practices (KAP) of health-related risks and behaviors among e-waste workers/recyclers belonging to the informal sector in Delhi and to determine the concentration of Pb levels in hair samples from a subset of workers from selected recycling sites.

**Methods.:**

A cross-sectional study was conducted in three e-waste informal recycling sites of Delhi: Seelampur, Mustafabad and Mandoli using an interviewer administered questionnaire among 220 e-waste workers. Percentages were presented for each KAP indicator. Analyses were computed using the statistical software STATA 14.2.

**Results.:**

It was observed that 24% of participants had knowledge of the meaning of e-waste and 36% knew the chemicals that could be released from e-waste handling. Personal protective equipment (PPE) was used by only 12% of the workers. Twenty-six percent (26%) perceived occupational injuries (cuts or burns) as mild and 20% perceived coughing immediately after work as mild. Explorative analysis showed a link between worker education level and the type of work practices employed. The median level of Pb concentration among hair samples was 8 mg/kg with inter-quartile range between 5.8 to 12.4 mg/kg.

**Conclusions.:**

Knowledge and practices among e-waste workers regarding the health hazards associated with e-waste recycling were poor with little knowledge of or attention to health risks. A comprehensive remediation package covering sensitization and awareness-building strategies of the health risks associated with informal e-waste recycling should be an urgent priority.

**Participant Consent.:**

Obtained

**Ethics Approval.:**

Ethics approval was obtained from the Institutional Ethical Committee (IEC), Indian Institute of Public Health-Delhi.

**Competing Interests.:**

The authors declare no competing financial interests

## Introduction

Electronic and electrical waste (e-waste) is defined as “any end-of-life equipment which is dependent on electrical currents or electromagnetic fields in order to work properly”.^1^ The most commonly used electronic items are batteries, mobile phones, laptops, computers, chargers, electronic home appliances, televisions and other similar devices.^1^ In 2012, e-waste production was approximately 45.6 million tons (Mt) in developed countries.^2^ About 80% of the total e-waste generated around the world is recycled in informal settings in Nigeria, Ghana, China, India and other developing countries.^3^ Moreover, globally, there are an estimated 2.3 million occupational-related deaths each year and work-related injuries and illnesses that have an economic cost of approximately four percent of the world's gross domestic product.^4^

The general practice for such end-of-life electronic items is usually to throw the items away along with domestic garbage which is then collected by waste pickers. Later, the items are sold to scrapyards or to recyclers where they are dismantled and the reusable materials are separated.^1^ During the entire process, hazardous heavy metals like lead (Pb), cadmium (Cd), chromium (Cr) and zinc (Zn) together with lithium (Li) and persistent organic pollutants like polybrominated diphenyl ethers (PBDEs), polybrominated biphenyls (PBBs), polychlorinated biphenyls (PCBs) and dioxins like polyaromatic hydrocarbons (PAH) are released.^1^ Recyclers are often working in these hazardous environments without any personal protective equipment (PPE).^5^ Studies have shown that chronic exposure to these hazardous chemicals could have long-term effects like disruption in endocrine, cardiovascular, reproduction, lung and growth functions.^6^ Traces of heavy metal elements are also found in the breast milk of mothers working in e-waste recycling.^6^ Furthermore, e-waste toxic effects can be transmitted across generations.^6–9^

E-waste recycling activities take place in both the ‘formal' and ‘informal' economic sectors. In the formal sector, the process of recycling of end-of-life electronic items takes place in safe environments and with proper safety equipment and PPE, as required under the respective government laws.^10^ In the informal sector, the processes for dismantling and incineration are illegal, unsafe and unauthorized.^10^ The entire process of e-waste collection, its transportation-segregation-dismantling- recycling and disposal is performed manually by laborers.^3^

India is the world's sixth largest economy and the fifth largest generator of e-waste, discarding 1.7 Mt of electronic and electrical equipment in 2014.^3^ It has an open market for electronic items with various brands and prices ranging from affordable to expensive. Much of this electronic equipment ends up being simply thrown away.^11^ Due to a large volume of imports, subsequent self-generation of e-waste and less developed formal recycling, India is facing a big challenge in the handling of e-waste. It is generated in large numbers from the public and private sectors, followed by households.^12^ Out of the total e-waste generated in the country, western India accounted for the highest contribution at 35%, while the southern, northern and eastern regions contributed up to 30, 21 and 14%, respectively. E-waste is usually sent to the informal sector where the components are manually removed. As a result, over one million people engage in manual labor in e-waste recycling operations in India.^13^ Common crude recycling practices include hammering, unscrewing and heating the printed circuit boards to remove components. In this crude handling of e-waste, hazardous chemicals are released to which workers are exposed through the skin, inhalation, and ingestion. There has been an increase in the concern about exposure to PBDEs, PBBs, PCBs and heavy metals which significantly affect workers' health as their working and living environments are highly contaminated with these pollutants.^13^

**Abbreviations**
*E-waste*: Electronic/electrical waste
*PPE*: Personal protective equipment
*PAH*: Polycyclic aromatic hydrocarbons

The E-waste Management Rules, 2015, issued by the Ministry of Environment, Forest and Climate Change (Government of India), mandates all producers of electrical/electronic items as well as recyclers, dismantlers and refurbishers to register with the Central Pollution Control Board (CPCB) and follow the guidelines provided.^14^ Producers, through a mandated extended producer responsibility, are required to take back their produced goods once they have reached end-of-life. Violation of any of these rules and contributing to adverse health and environment impacts is a punishable offense. Despite this, more than 90% of e-waste is still being handled by the informal sector in urban slums.^15^ The informal recycling sector employs mostly unskilled migrant labor coming from states like such as Uttar Pradesh, Bihar, Orissa and West Bengal.^3^ A large portion of informal e-waste workers are men, women and children of lower socio-economic strata, living in urban resettlement areas.^2^ These workers are engaged in crude dismantling of electronic items to earn their livelihood, putting them at risk of serious adverse health effects.^3^ Thus, the objectives of the study are to assess the knowledge, attitude and practices (KAP) of health-related risks and behaviors amongst e-waste workers/recyclers belonging to the informal sector in Delhi and to determine the concentration of Pb levels in hair samples from a subset of workers from selected recycling sites.

## Methods

A cross-sectional study was conducted in the north-eastern district of Delhi, include the Seelampur, Mandoli and Mustafabad areas, which form one of the rural zones of Delhi. The people residing in this area are primarily migrants from neighboring states of India such as Bihar and Uttar Pradesh. These migrants live in *jhuggis* (slum dwelling/huts usually made of mud and corrugated metal sheets) and many of them are engaged in self-employed activities such as cable stripping, manufacturing of decorative articles and shoe making in addition to being employed as industry laborers, painters, masons, daily wage laborers or auto-drivers. The labor is cheap and widely available,^16^ which make these areas a well-known hub of informal e-waste recycling units. Large markets are present in these areas for dismantling and repairing e-waste. The Mandoli industrial area is very well known for its incineration and scrapyard work. These tasks are carried out mainly by men, hence, such areas are male-dominated.^17^ The study was conducted from December 2018 to March 2019.

### Study participants

The study participants included male and female participants aged 18 years and above, working and residing in the given study areas. Participants who had severe illnesses or were pregnant were excluded from the study.

### Sampling method

A local non-governmental organization with a presence in the study area and with an established rapport with potential study participants was contacted to facilitate sampling. Thus, the sampling method employed was convenience sampling. An equal proportion of workers were sampled from Seelampur, Mandoli, and Mustafabad. Participants were recruited by visiting workers' shops with the help of a local contact. From the main road of the area, a lane of the road was selected a priority, and the first shop which was present at the intersection of the lane and the main road was identified as a starting point for the interviews, and then subsequent shops were visited sequentially until the required sample size was achieved. The workers were informed about the purpose and procedure of the study and only after consent, they were included as participants and interviewed.

### Sample size calculation

A study in Nigeria reported that the proportion of knowledge of the pathways of exposure to hazardous chemicals was 43%.^18^ Therefore, the expected proportion was taken as 43% (in the absence of any studies and estimates from India), an absolute precision of seven percent (7%), desired confidence level of 95%, and adjusted for a ten percent (10%) non-response rate. The sample size arrived at was 219 using the formula in Equation 1:

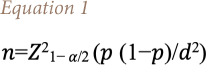
where p is the expected prevalence; d is the absolute precision; and Z^2^_1-a/2_ is the standard normal value for the desired confidence interval.

Additionally, for the determination of the Pb concentration level, hair samples were obtained from a feasible subset of the first 40 participants who provided consent.

### Study tools

An interviewer-administered questionnaire containing closed and open-ended questions was adapted as per the objectives and study setting and based on a similar study conducted in Nigeria (*Supplemental Material*).^18^ It was pretested in a group of five to seven e-waste workers. Knowledge responses were recorded as ‘Yes', ‘No’, and ‘Not Sure’. Attitudes were recorded as ‘None’, ‘Mild’, ‘Moderate’, and ‘Severe’. Practices were recorded as ‘Yes' and ‘No’. The study tool was translated and administered in the Hindi native language of the e-waste recyclers.

### Measurement of lead exposure

An approximate weight of five grams or 100–120 strands of human hair were obtained from 40 participants, from the occipital region of the scalp and 1.5 inches from the root in length. The hair was cut using scissors close to the scalp, so only the strands of hair above the scalp were tested and not the hair follicle. All 40 hair samples were sealed in airtight, clean and Pb free-plastic pouches separately. Hair that was dyed with color or henna was excluded. To maintain anonymity, the samples were coded with numbers.

The sealed hair samples were sent to the Shriram Institute of Laboratory Research, Delhi University, Delhi. The standardized process of washing, cutting, drying and analysis was performed by the laboratory.^19–21^ The hair samples were washed in Millipore water and acetone then dried at 100°C for 30 minutes. The samples were then weighed to 1 gram each and 10 ml digestion mixture consisting of 3 ml 69% nitric acid and 7 ml. Hydrogen peroxide (30%) was added. The prepared sample was further processed in the microwave digester and ml water was added to make 10 ml of the solution.^22–26^ To read the Pb levels, the laboratory used inductively coupled plasma-optical emission spectroscopy (ICP-OES) Vista-PRO.

### Analysis

The analysis was computed using the STATA version 14.2 software. The distribution of KAP indicators of e-waste workers related to adverse health risks were described using appropriate summary statistics. As an explorative analysis, the association of KAP indicators across socio-demographic factors (age, gender, education and type of work) and years of experience was assessed using multivariable logistic regression. As the responses were few in number and in order to obtain a more meaningful result, the sub-categories of ‘education' and ‘type of work' were merged as ‘no education', ‘primary' and ‘higher' and as ‘dismantler' and ‘non-dismantler' respectively.

In the knowledge category, four key indicators were taken for explorative analysis: meaning of e-waste, chemicals released from e-waste handling, routes of exposure, and knowledge about the names of PPE. The responses were categorized as ‘yes' and ‘no/not sure'. The Pb concentration was presented as in the form of mean and standard deviation.

### Ethics

Approval was obtained from the Institutional Ethical Committee (IEC), Indian Institute of Public Health, Delhi, India. Informed written consent was taken from the participants. To ensure anonymity of each participant, each questionnaire was coded with number identifiers only.

## Results

The analysis was completed on all 220 observations in the current study and there were no refusals. There were no missing values for any of the variables considered in the analysis.

**Figure 1:**
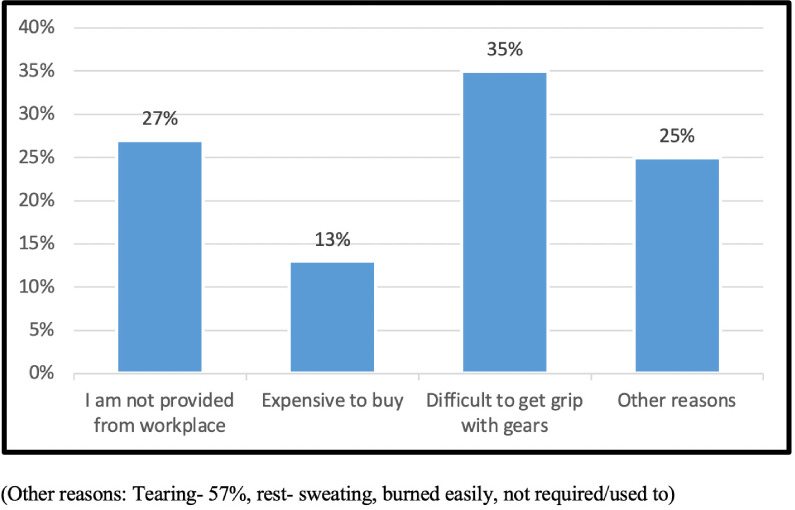
Reported reasons for not using personal protective equipment (n=193)

An equal number of participants were interviewed from all three areas of the study setting. The majority (80%) of the participants were male and illiteracy among the participants was high (46%) as shown in Table 1. About half of the e-waste workers interviewed were working as dismantlers. As shown in Table 2, 53 (24%) participants expressed knowledge of the meaning of e-waste. About 50% of the workers had knowledge about health care facilities available near to them. Twenty-seven (12%) reported the practice of wearing protective gloves at work *(Table 3).* Hand washing before going home or eating was practiced by 217 (98%) of the participants. Disinfectant hand wash and a clothes/utensil washing bar was used by 58 (27%) and 59 (27%) participants, respectively. As shown in Table 4, 81 (37%), 96 (44%), 67 (30%) and 15 (7%) participants had perceived injuries, including allergies/itching, coughing and stomach pain, respectively, rated from mild to severe (if any), immediately or within two to three hours of starting their work. Among those who did not use any PPE, the reported reasons are summarized in Figure 1. Sixty-six (35%) participants felt that it was ‘difficult to get a grip because of PPE' and 53 (27%) responded that ‘they were not provided with any at their workplace'.

**Table 1:** Socio-demographic Characteristics of Participants

***Characteristics***	***n (%)***

*Number of e-waste workers interviewed in each area*	
*• Seelampur*	74 (33.6%)
*• Mustafabad*	72 (32.8%)
*• Mandoli*	74 (33.6%)
*Age in years (mean* ± *SD)*	30 (±11.3)
*Gender*	
• *Male*	177 (80%)
• *Female*	43 (20%)
*Monthly family income in US Dollars (Median*, *IQ range)*	137 (102–164)
*Education*	
*• No education*	102 (46%)
*• Primary*	40 (18%)
• *Secondary*	63 (28%)
• *Higher than above*	15 (6%)
*Job description*	
*• Dismantler*	120 (54%)
*• Repairer*	16 (7%)
• *Torching*	25(11%)
• *Incineration*	13 (5%)
• *Acid washer*	1 (1%)
• Others*	45 (20%)
*Professional training received by e-waste workers*	0
*Years working in this profession (median, IQ range)*	5 (2–10)
*Number of days e-waste workers work in a week (mean* ± *SD)*	6 (±0.5)
*Mean (±SD) of hours e-waste workers work in a day*	9.2 (±1.8)
Number of workers working	
• Less than 9 hours a day	127 (58%)
• More than 9 hours a day	93 (42%)

*Others- segregation of metal components 14 (32%), smelting of aluminium 12 (27%), shredding of copper/iron manually 8 (17%), straining of aluminium/copper 8 (17%) and buffing/polishing 3 (7%).

**Table 2:** Distribution of Knowledge Indicators Among Participants of Health Risks

***Indicators***	***n (%)***

*Meaning of e-waste*	Yes: 53 (24%)
Chemicals/substances are released during dismantling/recycling of e-waste	Yes: 80 (36%)
If yes, then knowledge on following chemicals†:	
• Pb	9(11%)
• Copper	54 (68%)
• Lithium	1 (1%)
• Others (aluminium and iron)	31 (38%)
*On routes of exposure of chemicals interacting with the body*	Yes: 39 (18%)
If yes, then knowledge on following routes†:	
• Nose	37 (95%)
• Mouth	13 (33%)
• Open wound	2 (5%)
• Skin	8 (21%)
*Whether the chemicals/substances released from e-waste can affect their health*	Yes: 75 (34%)
*Whether they knew the name of any PPE:*	Yes: 62 (28%)
If yes, then knowledge of the following PPE†:	
• Gloves	60 (98%)
• Face/mouth mask	31 (50%)
• Eye-mask	0 (0%)
• Protective clothes	2 (3%)
*Knowledge of nearest health care facility*	Yes: 109 (50%)

† Percentages might not add up to 100, due to multiple responses,

**Table 3:** Distribution of Practice Indicators Among Participants Towards Health Risks

***Indicators***	***n (%)***

*Use any personal protective equipment*	Yes: 27 (12%)
*Washing their hand before going home/eating food?*	Yes: 217 (98%)
Use of the following for washing hands among those who report hand-washing (n=217)	
• Disinfectant hand wash	58 (27%)
• Washing powder	33 (15%)
• Clothes/utensil washing bar	59 (27%)
• Bathing soap	48 (22%)
• Plain water	19(9%)
*Have separate work clothes for their job*	Yes: 107 (49%)
Carry their clothes home regularly (for those who have separate clothes for work (n=107))	Yes: 49 (46%)
*Mode of management in case of any injury/illness:*	
*• None*	11 (5%)
• *Government*	87 (40%)
*• Private*	72 (33%)
*• Local healers*	37 (17%)
*• Home remedy*	11 (5%)

**Table 4:** Distribution of Attitude Indicators Among E-waste Workers Towards Health Risks

****Indicators****	***n (%)***			

	**None**	**Mild**	**Moderate**	**Severe**
*How do workers perceive injuries they experience?*	139 (63%)	58 (26%)	18 (8%)	5 (2%)
*How do workers perceive any allergy/itching immediately (2 to 3 hours of starting work)?*	124 (56%)	74 (34%)	17 (8%)	5 (2%)
*How do workers perceive any coughing immediately (2 to 3 hours of starting work)?*	153 (70%)	46(20%)	20 (9%)	1 (1%)
*How do workers perceive any stomach pain immediately (2 to 3 hours of starting work)?*	205 (93%)	9 (4%)	5 (2%)	1 (1%)
*Is the health of their family adversely affected by their occupation? (for example, work clothes, shoes, illness)*	No: 43 (20%)			

In Table 5, exploratory regression analysis using key knowledge indicators as the independent variables and knowledge of the meaning of e-waste was found to be associated with education. Compared to no education, those with a primary education had more knowledge about the meaning of e-waste (aOR=3.52; CI=1.39–8.32; p=0.021). Similarly, knowledge about the routes of exposure was associated with the type of work with borderline evidence of significance (p=0.069). Compared to dismantlers, those who did not work as dismantlers had better knowledge about the routes of exposure (uOR=1.97; CI=1.01–6.64).

**Table 5:** Crude and Adjusted Logistic Regression of Key Knowledge Indicators with Socio-demographic Factors, Type of Work and Years of Work Experience

***Knowledge on***	***Meaning of e-waste***		***Chemicals released due to handling of e-waste***		***Routes of exposure***		***Name any PPE***	
	**uOR (95%CI) *p-value***	**aOR (95%CI) *p-value***	**uOR (95%CI) *p-value***	**aOR (95%CI) *p-value***	**uOR (95%CI) *p-value***	**aOR (95%CI) *p-value***	**uOR (95%CI) *p-value***	**aOR (95%CI) *p-value***

*Age*	0.97	0.97	0.99	0.99	0.98	0.98	0.98	1.00
	(0.94–1.00)	(0.94–1.01)	(0.97–1.01)	(0.96–1.03)	(0.94–1.02)	(0.95–1.01)	(0.95–1.01)	(0.97–1.04)
	*0.091*	*0.299*	*0.695*	*0.965*	*0.505*	*0.365*	*0.272*	*0.696*
*Gender*								
• Male	1	1	1	1	1	1	1	1
• Female	0.94	1.72	0..92	0.84	2.06	1.54	0.43	0.44
	(0.43–2.07)	(0.69–4.27)	(0.45–1.85)	(0.35–1.77)	(0.79–5.36)	(0.68–3.48)	(0.18–1.02)	(0.17–1.14)
	*0.880*	*0.241*	*0.825*	*0.676*	*0.132*	*0.295*	*0.051*	*0.069*
*Years of experience*	0.98	0.99	0.98	0.98	0.96	0.96	0.97	0.97
	(0.94–1.02)	(0.94–1.05)	(0.94–1.02)	(0.94–1.03)	(0.91–1.03)	(0.91–1.01)	(0.93–1.01)	(0.92–1.02)
	*0.411*	*0.932*	*0.412*	*0.417*	*0.332*	*0.185*	*0.186*	*0.265*
*Type of work*								
• Dismantler	1	1	1	1	1	1	1	1
• Non–dismantler	0.38	0.43	2.33	2.42	1.97	1.94	1.37	1.22
	(0.18–0.71)	(0.26–0.88)	(0.80–3.69)	(0.76–3.65)	(1.01–6.44)	(0.96–3.92)	(0.62–2.99)	(0.54–2.74)
	*0.005*	*0.019*	*0.003*	*0.003*	*0.069*	*0.064*	*0.464*	*0.864*
*Education*								
• No education	1	1	1	1	1	1	1	1
	3.58	3.52	0.96	1.23	0.92	0.71	0.62	0.51
• Primary	(1.56–8.19)	(1.39–8.32)	(0.44–2.09)	(0.53–2.84)	(0.29–2.89)	(0.24–2.08)	(0.24–1.57)	(0.19–1.35)
• Secondary & above								
	1.98	1.89	1.46	1.55	1.54	1.39	1.72	1.50
	(0.95–4.11)	(0.84–4.25)	(0.79–2.69)	(0.79–3.01)	(0.68–3.49)	(0.65–2.94)	(0.91–3.27)	(0.75–2.98)
	*0.009*	*0.021*	*0.394*	*0.435*	*0.495*	*0.433*	*0.065*	*0.086*

aOR=adjusted odds ratio; uOR- unadjusted odds ratio

As shown in Table 6, there was a significant association between the practice of using PPE with the type of work, and those who did not work as dismantlers reported better practices around use of PPE compared to dismantlers (aOR=3.20; CI=1.12–7.84; p=0.011).

**Table 6:** Crude and Adjusted Logistic Regression of Use of Personal Protective Equipment with Socio-demographic Factors, Work and Years of Work Experience

***Use of PPE***	***Unadjusted OR (95%CI)***	***P-value***	***Adjusted OR (95%CI)***	***p-value***

*Age*	0.96 (0.91–1.00)	0.042	0.97 (0.92–1.02)	0.345
*Sex*				
• Male	1		1	0.162
• Female	0.29 (0.06–1.30)	0.102	0.32 (0.06–1.58)	
*Years working*	0.93 (0.87–1.01)	0.051	0.95 (0.87–1.04)	0.29
*Type of work*		0.022		0.011
*•* Dismantler	1		1	
• Non-dismantler	2.77 (1.15–6.33)		3.20 (1.12–7.84)	
*Education*				
• No education	1	0.302	1	0.591
• Primary	0.67 (0.17–2.54)		0.66 (0.16–2.71)	
• Secondary/Higher	1.65 (0.69–3.92)		1.31 (0.51–3.36)	

### Hair sample analysis

Eleven samples had Pb levels below the detection limit, hence their levels could not be obtained. The median level of Pb concentration among the remaining 29 samples was 8 mg/kg (IQR 5.8 to 12.4 mg/kg). The reference values across worldwide studies are given in Table 7.

**Table 7:** Concentration of Lead Levels Across Worldwide Studies

***Study Site***		***Lead level (mg/kg)***

*E-waste recycling site, Delhi, India (Present study)*	Median (Range)	8 (5.8–12.4)
*European Reference Material, European Reference Materials consortium* 27	Mean (±SD)	2.1 (±0.2)
*E-waste recycling site in slum in Bangalore, India* 28	Geometric mean(Range)	9.1 (2.4–74.5)
*Reference site in Chennai, India* 28	Geometric mean (Range)	2.6 (0.9–19.8)
*Waste disposal site in Perungudi, India* 29	Median	28.1
*Reference site in Palaverkadu, India* 29	Median	11.4
*Suburban residents of Gia Lam, Hanoi, Vietnam*^30^	Median	4.99
*Suburban residents of Thanh Tri, Hanoi, Vietnam*^30^	Median	9.83
*Reference value in children, Czech Republic* 31	Median	1.96
*Reference sites taken from Canada, USA, Poland, Japan and India* 32	Mean	5.38, 5.35,2.52, 3.62, 13.2
*Standard reference material, Institute National de la Sante Publique—Laboratoire de Toxicologie, Quebec, Canada* 33	Mean	4.1
*Reference sites from USA* 34	Geometric mean	2.4
*Reference site Taif city, Kingdom of Saudi Arabia* 21	Mean	5.0
*Progression of trace element levels in diabetes patient and hypertensive patient*^21^	Mean (±SD)	7.01 (±0.38); 9.12 (±1.16) ^[21]^
*Reference values from children, Rome, Italy*^20^	Median (Range)	5.60 (1.0–19.8)
*Workers of a Pb refinery industry, Zanjan, Iran* 35	Mean (±SD)	131.7 (±93.4)

## Discussion

The present study revealed that the knowledge of e-waste workers across the indicators studied was poor, their working practices put them at high risk, and they did not perceive significant detrimental health threats (as a result of their exposure to e-waste) towards themselves or their families. There was some evidence to suggest that their education and type of work might influence their knowledge of possible health risks.

A study in Nigeria and Ghana reported poor knowledge and health practices, where 88% of e-waste workers did not know the name of any chemical present in e-waste and only 18% used PPE.^18,36^ A study in Hyderabad, India reported that a far lower proportion of informal e-waste workers had any knowledge about health risks, as 72% of waste handlers did not know the meaning of e-waste and 71% were not aware of any associated adverse health risks.^12^ A systematic review reported that the high workload on e-waste workers and extreme poverty might lead to their poor knowledge.^37^ In the present study, the majority of e-waste workers did not know the meaning of e-waste, although they worked on end-to-life electronic products. Their responses to questions on the names of the chemicals were in line with the type of work with which they were involved. For example, it was observed that in Seelampur and Mustafabad, the lanes of the road were divided by type of work—dismantling and straining/shredding of copper. As a result, workers in these areas were aware of metals like copper and Pb. Dismantlers believed that segregation of electrical components did not release chemicals and the processes did not involve any burning of components, and therefore it was safe and would not affect their health.^38,39^

The International Labour Organization (ILO) reported that globally, a huge percentage of e-waste workers are engaged in high risk practices without any PPE.^39^ The present study also reported that very few e-waste workers were using PPE, with the most commonly reported PPE being gloves made of cloth or wool. Workers involved with smelting and torching reported that gloves get burned easily and dismantlers reported that it was difficult to get a grip (with hammers, screwdrivers and chisels) with the gloves they had. This information was also in line with findings in other systematic reviews.^1,5,13^ Forty percent (40%) of e-waste workers had perceived their symptoms of coughing/itching/allergies and injuries as mild to moderate (if any) but they did not perceive these problems as being attributable to their occupation. There is no study which directly relates these symptoms to e-waste in India, but a case report by Jayapradha Annamalai in 2015 reported the incidence of occupational health hazards such as respiratory/skin disorders from the processes associated with informal recycling of e-waste in India.^40^ At the time of any injury, most e-waste workers prefer to go to non-governmental facilities such as private/local healers, so as to save time and wages.

Studies in Nigeria, Hyderabad and Delhi reported that the factors which could be associated with poor knowledge towards adverse health risks were education and type of work.^12,18,38^ This was similarly observed in the present study, where less education and a certain type of work (dismantler) was associated with poor KAP indicators towards occupational health risks. Only one practice indicator was found to be associated with the type of work, and that was a higher number of non-dismantlers as compared to dismantlers reported using PPE.

The mean Pb concentration level in a subset of the hair samples was found to be 8 mg/kg (IQ range 5.8 to 12.4 mg/kg). This level was slightly higher than the reference value of 5 mg/kg from studies completed elsewhere in the world,^21^ but comparable to that in a study in Bangalore (over 9 mg/kg, IQ range 2.3 to 73.4 mg/kg) in 14 samples in e-waste recycling slums.^28^ These levels might indicate human exposure to Pb from e-waste recycling sites, which does however depend on the methods used in those facilities for recycling like dismantling, smelting, or torching.^28^ A study in Romania of two case groups (one exposed to a high concentration of Pb in air and the other to lower concentration) and a control group (unexposed) of male workers with different occupational experiences indicated that the Pb content of hair in occupationally exposed workers was significantly correlated with blood lead levels (BLL).^41^

The strength of the present study was that it is the first to explore e-waste workers' levels of awareness of health risks in this study area. In addition, the study was conducted on an adequate sample size, which made the results meaningful. Various insights on the perceptions and attitudes towards health amongst the informal e-waste recyclers were obtained. These findings can help to design programs for appropriate remediation and rehabilitation of informal e-waste recyclers.

Certain limitations of the present study should be considered when interpreting the results. A non-probability sampling (convenience) method was used, and therefore the ability to generalize the study findings is restricted. The questionnaire was translated and administered in the local language (Hindi). Back-translation to the English version to check accuracy was not performed, although translators and interviewers well-versed in both languages were involved, limiting any concerns in the interpretation of the findings. The association between socio-demographics and KAP indicators was part of an exploratory analysis, hence the results should be cautiously interpreted. Lead levels found in hair samples were studied in only in a subset of primary samples, hence no conclusive interpretation can be made. Because the hair samples were taken from a feasible subset, this could have resulted in sampling bias. Furthermore, dyed hair was excluded from hair sampling, which might have led to gender imbalance as women are more likely to color their hair. A comparative study with a control group not involved in e-waste recycling would have helped to compare associations between e-waste handling and Pb exposure levels, but budgetary constraints precluded this. A correlation with BLLs might also have been of additional value. Furthermore, information on other important covariates such as smoking, drinking, shampoo, and other environmental exposures was not explored given the scope of the present study. However, some associations have been observed in the literature between smoking and Pb content in hair. In a study from Poland, a positive correlation was found between Pb in hair and smoking (2.24 ± 3.05 mg/kg)^42^; in the study from Lithuania, the geometric mean of Pb in hair adjusted for age, smoking, intake of strong alcohol, and hair dying of occupationally exposed workers (7.6 mg/kg) was significantly higher than that in those not exposed to Pb (3.2 mg/kg)^43^; in a study from Jordan, the mean concentration of Pb in hair samples of male smokers was 11 mg/kg.^44^

## Conclusions

E-waste management is an emerging environmental issue requiring cooperation among all stakeholders. Delhi, the capital of India, has become the capital of e-waste generation in India. Even after the commencement of e-waste management rules in 2011, the amount of e-waste generated continues to increase, along with the number of informal workers involved in e-waste recycling. Workers put their health at risk with direct exposure to e-waste due to infrequent usage of PPE. Informal workers often underestimate the health risks associated with their occupation. It is important to make workers in these informal settings aware of potential health-risks.

In line with the actions of the CPCB and the Delhi government to reduce the e-waste burden and informal e-waste recycling practices, the authors of the study put forward several recommendations. Efforts should be made to increase the awareness level of workers, through counselling or meetings in their community to share knowledge about the hazards associated with improper e-waste handling. The owners of e-waste recycling units should be incentivized to make the use of PPE mandatory. Owners and workers should also be trained to pursue formal recycling processes under the CPCB directives to reduce potential health risks and hazards with due attention to possibilities for rehabilitation and alternate livelihood options for these informal recyclers.
